# Obstetric rheumatology training is an unmet educational need within the rheumatology community

**DOI:** 10.1093/rap/rkaa025

**Published:** 2020-07-14

**Authors:** Iona Thorne, Kokul Sriskandarajah, Maria Mouyis

**Affiliations:** Department of Rheumatology, Chelsea and Westminster NHS Foundation Trust, London, UK

**Key message**

- Obstetric rheumatology training is an unmet educational need within the rheumatology community.

Dear Editor, Inflammatory rheumatic diseases have a predilection for women and disease onset that often overlaps with peak reproductive life. Maintenance of disease control in pregnancy is vital because active disease is associated with adverse pregnancy outcomes, and predictors of adverse outcomes in pregnancy are lacking [1]. The on-going maternal mortality surveillance programme (MBRRACE-UK) often reveals how the impact of poor family planning, pre-pregnancy planning and antenatal care can be catastrophic and recommends that it is seen as the responsibility of all health professionals to facilitate opportunistic pre- and post-pregnancy counselling and appropriate framing of the advice when women with pre-existing conditions attend any appointment [2].

People with inflammatory rheumatic disease have described an unmet need for access to family planning, pre-pregnancy counselling and specialist management around pregnancy [3] and report difficulty accessing adequate information on pregnancy planning, pregnancy and early parenting [4]. Clinical practice in this area has also been shown to be very variable [5].

In April 2019, ∼250 rheumatology health-care professionals attended the inaugural meeting of the British Society for Rheumatology’s Special Interest Group for Pregnancy and Rheumatic Disease, where baseline knowledge and educational need were explored. A questionnaire was distributed exploring pre-pregnancy counselling for women with rheumatic disease and the management of complications of rheumatic disease during the antenatal and postpartum period. Confidence with a variety of clinical issues was explored using a 10-point Likert scale.

Eighty-four questionnaires were fully completed and returned, predominantly by rheumatology clinicians working in secondary and tertiary care settings. All reported seeing women of childbearing age with rheumatological conditions in their clinical practice and, for the majority, counselling about contraception (84%), fertility (64%), pre-pregnancy counselling (90%) and breastfeeding (83%) formed part of their usual clinical practice. Many, however, reported that they have not received any formal training in reproductive aspects of rheumatology (20%) and have learnt by attending a lecture (48%) or courses (10%). Commonly expressed areas of unmet educational need were treatment decision-making about rheumatic disease in pregnancy, post-natal rheumatic issues, contraception in rheumatic disease and the effect of hormones and fertility treatment on rheumatic disease. Only 24% had experience of using the UK Teratology Information Service.

It is clear that there is an unmet educational need within the rheumatology community to ensure that all rheumatology health professionals are trained to facilitate opportunistic pre- and post-pregnancy counselling at any appointment and to ensure that our patients can access family planning and specialist management around pregnancy. Such training is essential to improve health outcomes (for both mother and baby), address the currently unmet needs expressed by our patients and improve access to specialist services.

The British Society of Rheumatology’s Special Interest Group for Pregnancy and Rheumatic Disease are committed to promoting and facilitating educational opportunities, and we welcome collaboration to achieve this aim.

*Funding:* No specific funding was received from any funding bodies in the public, commercial or not-for-profit sectors to carry out the work described in this manuscript.

*Disclosure statement:* I.T. and M.M. are convenors and members of the BSR Special Interest Group for Pregnancy and Rheumatic Disease.

## References

[1] de Man YA, Hazes JM, van der Heide H et al Association of higher rheumatoid arthritis disease activity during pregnancy with lower birth weight: results of a national prospective study. Arthritis Rheum 2009;60:3196–206. 19877045 10.1002/art.24914

[2] Knight M, Bunch K, Tuffnell D et al (eds), on behalf of MBRRACE-UK. Saving lives, improving mothers’ care – lessons learned to inform maternity care from the UK and Ireland Confidential Enquiries into Maternal Deaths and Morbidity 2014–16. Oxford: National Perinatal Epidemiology Unit, University of Oxford 2018.

[3] Patient engagement data from Versus Arthritis and Lupus UK (unpublished).

[4] Ackerman IN, Jordan JE, Van Doornum S, Ricardo M, Briggs AM. Understanding the information needs of women with rheumatoid arthritis concerning pregnancy, post-natal care and early parenting: A mixed-methods study. BMC Musculoskelet Disord 2015;16:194. 26285693 10.1186/s12891-015-0657-4 PMC4545539

[5] Panchal S, Khare M, Moorthy A et al Catch me if you can: a national survey of rheumatologists and obstetricians on the use of DMARDs during pregnancy. Rheumatol Int 2013;33:347–53. 22451029 10.1007/s00296-012-2418-0

